# WM in Adolescence: What Is the Relationship With Emotional Regulation and Behavioral Outcomes?

**DOI:** 10.3389/fpsyg.2018.00844

**Published:** 2018-05-29

**Authors:** Chiara Malagoli, Maria Carmen Usai

**Affiliations:** Department of Education, University of Genoa, Genoa, Italy

**Keywords:** working memory, emotional regulation, behavioral outcomes, adolescence, individual differences

## Abstract

Adolescence is a fundamental transition phase, marked by physical, social, cognitive and emotional changes. At this stage in development two contrasting phenomena take place: brain changes cause a sensitivity to emotional aspects (Dahl, 2004); while also control processes register as well impressive improvements (e.g., Hooper et al., 2004; Best and Miller, 2010). The study is aimed to investigate the relationship between a core cognitive feature such as working memory (WM) (Diamond, 2013) and complex abilities such as emotion regulation (ER) and behavioral self-reported outcomes using a structural equation model approach. A sample of 227 typically developed adolescents between 14 and19 years of age (148 females; mean age in months 202.8, SD 18.57) participated in this study. The following tasks and self-reports were administered in a 45-min test session at school: Symmetry Span task (Kane et al., 2004). Reading Span task (Daneman and Carpenter, 1980), Mr. Cucumber (Case, 1985); Youth Self-Report (YSR, 11–18 years, Achenbach and Rescorla, 2001); Difficulties ER Scale (DERS, Gratz and Roemer, 2004; Italian version by Giromini et al., 2012). Results showed that difficulties in ER correlated with WM: high levels of ER difficulties are associated with low WM efficiency while no significant contributions of these predictors was observed on externalizing or internalizing symptoms. This study showed a significant relationship between self-reported difficulties in ER and WM, while no significant contribution of the considered predictors was showed on the outcomes, adding knowledge about how behavioral and emotional self-reported outcomes may relate to these processes.

## Introduction

Adolescence is a special time in development. Dahl (2004) defines adolescence as “*The developmental interval that encompasses the body and brain changes of puberty*.” During this time, two apparently contrasting developmental phenomena occur. On one hand, brain changes cause a sensitivity to emotional aspects of experiences that influence the increase in emotional arousal and such phenomena as sensation-seeking, risk-taking, increased conflict with parents, increased mood volatility and a particular increase in negative emotions (Dahl, 2004). On the other hand, cognitive processes, particularly cognitive control functions, register impressive improvements exhibited in such abilities as abstract thought, organization, decision-making, planning, rule management, and flexible adaptation to different contexts (Johnson, 2000; Anderson et al., 2001; Hooper et al., 2004; Best and Miller, 2010). These developmental outcomes are apparently in opposition to each other because the ability to generate plans and reasoning in the abstract would not explain risk-taking, sensation-seeking and recklessness or simply general impulsiveness that is typically observed during adolescence. This situation is particularly interesting, since adolescents seem to have all of the crafts necessary to evaluate correctly and plan actions. Many aspects of decision-making appear adult-like, but adolescent decisions may still not be coherent, and as a consequence, it is possible to observe mal-adaptive or even dangerous behaviors.

In the literature, most of the studies on adolescence seem to be more focused on extreme clinical outcomes, such as conduct disorders (e.g., Kim et al., 2001), drug and alcohol abuse (see for a review Peeters et al., 2015; Kim-Spoon et al., 2017), and aggressive behaviors, such as bullying (e.g., Hamama and Ronen-Shenhav, 2013; Pouwels et al., 2017), while fewer investigations have concerned typical adolescence and the impact of superior cognitive features, such as working memory (WM), on the complex relationships between difficulties in emotion regulation (ER) and behavioral outcomes, with both being considered not as clinical issues but as normally challenging features of adolescence. The present study aims to try to fill this gap in the literature by investigating these aspects in a typical population of adolescents, with a particular attention to individual differences in difficulties in ER, WM efficiency, and behavioral outcomes.

### Emotion Regulation During Adolescence

During adolescence, everyday life situations characterized by strong affective stimuli, as mentioned, often result in enhanced emotional outcomes. Although adolescents present a more mature and perfected awareness of emotions, in comparison to children, in general, the control functions exhibited by adolescents often emerge to be unsatisfactory (Casey et al., 2008). This phenomenon has been addressed by examining a variety of possible developmental reasons, such as hormonal activation, different brain development in regions that underlie this imbalance (Giedd et al., 1996, 1999; Sowell et al., 1999; Gogtay et al., 2004; Barnea-Goraly et al., 2005) and, in particular, elevated activity in the ventral striatum observed during adolescence (Ernst et al., 2005; Galvan et al., 2006; Delgado, 2007; Van Leijenhorst et al., 2010; Sescousse et al., 2013), which seems to influence such processes as risk-taking and decision-making.

Moreover, adolescence is a period in which the peer group became more central and also friendship acquires importance (Rubin et al., 2008). In this perspective, the open question is whether failures in ER or dangerous behaviors and Rule-Breaking may be due to a specific difficulty in regulating emotions and behaviors or if being risky and impulsive may also be mediated by individual and specific features that may or may not also be elicited by the social environment. In terms of ER, defined as the process that elicits the onset, offset and magnitude/duration or quality of one or more emotional features of emotional response (Gross, 1998; Gross and Thompson, 2007), more or less adaptive abilities may also be linked to individual differences in more cognitive forms of ER. In fact, less efficient forms of this specific type of regulation have been connected to poor psychological well-being (Balzarotti et al., 2016). Indeed, as reported by Ahmed et al. (2015), this specific period of life was found related to an enhanced occurrence of internalizing and externalizing problems (Spear, 2000; Paus et al., 2008; Lee et al., 2014). This finding suggests that adolescents may be vulnerable to emotional dysregulation, which may cause not only maladaptive outcomes but also may affect cognitive processes, including WM, that undergo development during adolescence (Somerville and Casey, 2010; Sebastian et al., 2011; Blakemore and Robbins, 2012; Dumontheil, 2014) and may be particularly challenged in more complex every-day life situations.

### Working Memory in Adolescence

Working memory refers to a system that can maintain and process information simultaneously (Engle et al., 1992; Oberauer et al., 2016). WM is also defined as the ability to maintain representations of recently experienced or recalled information over a short period of time (Curtis and D’Esposito, 2003). Therefore, WM is required for the optimal performance of goal-directed behaviors (Diamond, 2013; for a discussion Morra et al., 2018). Important changes occur in WM during development, and WM task performance and latent structure have been shown to evolve throughout middle and late adolescence (Malagoli and Usai, 2018) following a protracted course of development into young-adulthood (Huizinga et al., 2006). Individual differences in WM capacity are observed to be correlated with a variety of cognitive and social outcomes, including school performance (Gathercole et al., 2004; Dumontheil and Klingberg, 2012; Finn et al., 2014). Research has shown that WM filtering ability – the ability to filter extraneous or distracting information from WM during encoding – is strongly associated with overall WM capacity and accuracy (Vogel et al., 2005; Peverill et al., 2016).

### Which Relationship Between WM and ER?

Considering WM specifically, many studies run in laboratories have reported that WM performance is affected by emotionally based stimuli both in a positive and negative way, such as a more vivid memory for emotional pictures (Canli et al., 2000), emotional word-lists (Jones et al., 1987; Dietrich et al., 2001), or humor (Schmidt and Williams, 2001), while there are other situations in which the most adaptive behavior is actually ignoring emotional information and attempting to not be affected by it.

Romer et al. (2009, 2011) performed a large cohort study investigating WM ability in a sample of early adolescents (*n* = 387, ages 10–12 at baseline). In three annual assessments, these researchers examined models in order to understand the trajectory of weak WM, early manifestations of externalizing problems, and heightened levels of trait impulsivity. Participants were tested with a computerized battery of tasks to assess WM, cognitive control, and reward processing, plus an audio-guided computerized self-interview for impulsivity and risk behaviors and a self-report questionnaire to evaluate externalizing and internalizing difficulties (YSR Achenbach and Rescorla, 2001). Romer et al. (2009) study found WM only indirectly related to externalizing behavior due to its relationship with acting without thinking. In a second study (Romer et al., 2011) with the same sample, the authors found that WM prospectively seemed to predict reduced externalizing behavior both directly and indirectly, with a mediation effect by acting without thinking. In addition, WM positively predicted sensation seeking, which was positively related to externalizing behavior also controlling for acting without thinking. These research suggests that WM may have a positive relationship with externalizing behavior with a mediation of more complex aspects as sensation seeking.

These studies show the importance of WM in particular as a cognitive factor that can relate not only to behavioral outcomes but that may also explain more emotional forms of regulation, such as sensation-seeking and risk-taking. However, these studies do not address another research question of how WM may be related to specific difficulties in ER in this sensitive time of development.

### Driven or Non-driven Behaviors, WM and ER: What Are the Implications in Everyday Life?

ER comprehends both more automatic features and more effortful ones: the ability to voluntarily guide behavior and make an effort to direct the flow of thoughts and emotions in a goal-directed way is essential to mature decision-making, while more automatic forms of ER are important and useful for directing the flow of emotions and thoughts in more known situations. From this perspective, Gyurak et al. (2011) state that implicit processes may be evoked in an automatic way by the stimulus itself and completed without an active monitoring. Cognitive control processes, such as WM, allow us to voluntarily guide our behavior and support both forms but particularly explicit forms of regulation need this kind of cognitive control. While adolescents can demonstrate refined voluntary behavior, the ability to maintain consistently this attitude continues to improve during adolescence, for this reason cognitive control features are particularly interesting to investigate the vulnerabilities of this period. In this sense, and in particular at this age, ER may have a fundamental role in controlling impulses and behaviors. The ability to manage emotions, as mentioned, is a relatively voluntary, effortful and deliberate process, that attempts to outbalance more spontaneous emotional responses. Finally, ER allows people to enhance, maintain, or reduce both negative and positive emotions. Coherently with these features, ER often implies some adjustments in emotional responding. Ironically, these emotional adjustments may not reach the individual’s goal of a particular emotional state (e.g., trying to switch from anxious to calm), and these “defeats” may also mirror strong and emotional outcomes that people would usually like to disguise (Wegner et al., 1993) or exhibit the emotions they actually wanted to hide regardless their efforts. In these specific circumstances, the natural salience of emotional stimuli and the human tendency to process them transform these episodes into strong interferences in competition for cognitive resources with more relevant information (Ellis and Ashbrook, 1988), often resulting in decreasing performance on the task in action (Dolcos and McCarthy, 2006; Dolcos et al., 2008; Anticevic et al., 2010; Chuah et al., 2010; Denkova et al., 2010). Considering the enhanced emotional arousal documented in adolescence (Dahl, 2004), investigating how difficulties in emotional regulation may be related to WM may be particularly useful in order to better understand the vulnerabilities of this developmental stage.

## The Present Study

The data illustrated in this paper are part of a larger study investigating cognitive processes in adolescence (Malagoli and Usai, 2018). The aim of this study is to explore the relationship between specific self-reported difficulties in ER, WM and behavioral outcomes, adding knowledge regarding this topic and contributing to the analysis of individual differences in typical development. Due to the importance that WM has in predicting stronger regulation abilities (e.g., rule management, updating of useful information) (Diamond, 2013) and the prolonged development in time that both WM and the ability to regulate emotion show (Dahl, 2004; Huizinga et al., 2006), we expect difficulties in ER are associated with WM performance (Dietrich et al., 2001; Jones et al., 1987) and to be related with behavioral outcomes (Anticevic et al., 2010). We expect also WM to be related to specific behavioral outcomes (Dahl, 2004; Dolcos and McCarthy, 2006; Dolcos et al., 2008; Anticevic et al., 2010; Chuah et al., 2010; Denkova et al., 2010).

## Materials and Methods

### Participants

A sample of 240 14- to 19-year-old adolescent (158 females) high school students participated in this study. The participants were excluded if they did not speak Italian as their first language or had been diagnosed with any disease (e.g., learning disabilities) or neurological (e.g., brain infection) or psychiatric disorder. Eight participants were excluded due to learning disabilities, four were excluded for not having Italian as their first language, and one was excluded due to neurological issues. The final sample included 227 participants (148 females; mean age in months 202.8, SD 18.57).

### Materials and Procedure

We administered one 45-min test session in a quiet room that was provided by the school. A symmetry-span task, reading span task and the Mr. Cucumber task were administered by a trained experimenter. The task sequence was exactly as listed above. Questionnaires were asked to be filled out during the session, and participants were asked to return them at the end of the administration day.

#### Self-Report Measures

*Difficulties in Emotion Regulation Scale* (DERS, Gratz and Roemer, 2004; Italian version by Giromini et al., 2012). The DERS is a self-report questionnaire composed by 36 items developed to assess severe difficulties in ER abilities. Scores are provided for six scales: Non-acceptance of Emotional Responses (Non-acceptance, 6 items), Difficulties Engaging in Goal-Directed Behavior (Goals, 5 items), Impulse Control Difficulties (Impulse, 6 items), Lack of Emotional Awareness (Awareness, 6 items), Limited Access to ER Strategies (Strategies, 8 items), and Lack of Emotional Clarity (Clarity, 5 items). Participants may set their responses on a 5-point Likert scale ranging from 1 (almost never) to 5 (almost always). To determine the internal consistency of the questionnaire, Cronbach’s alpha was calculated for the total DERS score and for each subscale. Cronbach’s alphas were larger than 0.70 for all of the scales: Non-acceptance 0.73, Goals 0.85, Impulse 0.85, Awareness 0.72, Strategies 0.89, Clarity 0.84 and DERS Total 0.92.

*Youth Self-Report* (YSR, 11–18 years, Achenbach and Rescorla, 2001, Italian version as available on the http://www.aseba.org website). This questionnaire is a screening measure for behavioral and emotional difficulties in children and adolescents. The YSR is also part of the Achenbach System of Empirically Based Assessments (ASEBA). The 2001 revised YSR comprises 112 items in a six-month time lapse. Participants are asked to indicate how often a certain behavior applies to them on a three-point scale (0 = absent, 1 = occurs sometimes, 2 = occurs often). Scores are provided on eight subscales: Anxious/Depressed, Withdrawn/Depressed, Somatic Complaints, Social Problems, Thought Problems, Attention Problems, Rule-Breaking Behavior, and Aggressive Behavior. Subscales are clustered in order to identify individual’s externalizing or internalizing profiles. Internalizing is the resulting profile from Anxious/ Depressed, Withdrawn/Depressed, and Somatic Complaints scores, and Rule-Breaking Behavior and Aggressive Behavior result in the Externalizing profile. Cronbach’s alphas were larger than 0.70 for all the scales: Anxious/Depressed 0.74, Withdrawn/Depressed 0.75, Somatic Complaints 0.73, Social Problems 0.71, Thought Problems 0.72, Attention Problems 0.73, Rule-Breaking Behavior 0.73, and Aggressive Behavior 0.70.

#### Working Memory Tasks (for an Extensive Description See Malagoli and Usai, 2018)

*Symmetry span task* (SymmSpan; Kane et al., 2004). This task is a complex measure of WM capacity composed of two different tasks that are performed at the same time. The first task consisted of recalling a sequence of squares that turned red on a matrix that appeared on the screen, while the second consisted of judging figure symmetry. The tasks were clustered together in two to five sets. Every square presentation was spaced out by a symmetry task. For example, if a set is composed of two sets, two squares and two symmetry problems, alternatively, all of the squares must be recalled at the end of each set. A 4×4 square matrix appeared in the center of the screen, and one of them turned red. Then, the symmetry judgment task was presented in an 8×8 matrix, with some squares filled in black, and the participants decided whether the black-square design was symmetrical along its vertical axis. Set sizes ranged from two to five symmetry–memory matrices per trial (for 12 trials total). The presentation of sets is sequential both for the square and symmetry problems. The participants were instructed to recall the whole sequence of squares in the correct order and to maintain at least 85% accuracy on the symmetry trials. Three controls appeared on the screen, and they were available to use during the participants’ recall: “blank” to point to a square that they could not recall, “clear” to delete the sequence and attempt it again, and “exit” to go to the next set. These controls were activated by the participants themselves using a touchpad. The participants had an unlimited amount of time to recall all of the squares. The computer calculated the mean RT of the participants in the practice phase for the symmetry problems to use in the test phase. The mean RT was expressed to the participants after the practice phase. Feedback was provided at the end of each set, informing the participants of their performance accuracy. One practice block was presented for the square task (four square sets), one for the symmetry task (15 symmetry problems) and one for the combined task (three sets with three square tasks and three symmetry problems). The test phase was composed of three sets of three combinations, three sets of four combinations and six sets of five combinations. The dependent variable was the absolute span score, which was computed using the traditional absolute span scoring method. This score was the sum of all perfectly recalled sets. For example, if an individual correctly recalled two squares in a set of two, three squares in a set of three, and three squares in a set of four, their SPAN score would have been five (2 + 3 + 0). A split-half reliability procedure was performed for this task. The Spearman-brown coefficient was 0.91.

*Reading span task* (RSPAN; Daneman and Carpenter, 1980). This span task is structurally identical to the previous one but with different stimuli. The task consisted of recalling a sequence of letters that appear on the screen while the participants judged whether some phrases made logical sense. The two tasks were clustered in sets (ranging from two to seven). Every letter in each set was spaced out by a phrase problem. The participants were asked to recall the entire sequence of letters in the correct order at the end. The letters appeared in the center of the screen one by one. The sentences also appeared written in the center. Each sentence consisted of 10–15 words. Letter practice used sequential selection. The letter-sentence practice and the letter-sentence test used random selection without replacement for each sequence. When presented with the recall cue, the participant recalled each letter from the preceding set, in the order in which they appeared, by selecting them using the touchpad from a matrix of 12 alternatives. The set sizes ranged from two to five sentence–letter problems per trial (for 12 trials total). The three controls (“blank,” “clear,” and “exit”) were kept the same. The participants were given all of the time that they needed to recall the letter sequence. The computer calculated the participants’ mean RT during the sentence problem practice phase, and this personalized mean time was the maximum time that the participants had to solve sentence problems during the test phase. Feedback was also provided at the end of each phase. The phases were the same, and they consisted of letters (two sets of two and two sets of three), phrase practices (15 letters), and combined practices (two sets of two and three sets of three letter/phrase combinations). Finally, a combined test phase that was composed of three sets of three, four, five, and seven letter and/or phrase combinations was administered. The participants received feedback as they did in the previous task. The participants were instructed to try to be precise in recalling the letter sequence and to attempt to maintain at least 85% accuracy in judging the sentences. The absolute span score (i.e., the sum of all of the perfectly recalled sets, RSPAN) was used. A split-half reliability statistic was calculated for this task. The Spearman-brown coefficient was 0.91.

*Mr. Cucumber task* (Case, 1985). This task is a classic visual-spatial span measure. The outline of a complex figure (an extraterrestrial) was presented, to which colored stickers were applied. This non-computerized task comprised three practice items and a test phase with eight levels. In each one, three items were displayed. The subjects were able to watch the figure for 5 s until the fifth level, and the stickers appeared for the last three levels. The task required the participants to recall the position of all of the stickers by pointing to a figure without a sticker. A thick sheet of paper depicting a grill was shown to avoid any contribution of iconic memory when watching the time lapse and before presenting the recall figure. A point was given for each level that was fully correctly recalled. One-third of a point (0.33) was given for each correct item beyond that level. The test was discontinued if the participants failed on all three items in the same level. The dependent measure was the score that was obtained (expected range 0–8). The Cronbach’s alpha was 0.88.

### Data Analysis

Descriptive statistics (i.e., means, standard deviations, possible score ranges, skewness and kurtosis) and zero-order and partial (Pearson) correlations controlling for age were calculated. Outlier values that deviate from the mean more than three standard deviations were excluded from the analyses. In addition, 9 values were excluded from memory scores because they did not maintain at least 85% accuracy. The total excluded values representing 1.8% of the full sample. A series of Structural Equation Models (SEM) were conducted based on raw data using MPlus 7.4 software (Muthén and Muthén, 1998–2010). The maximum likelihood with robust standard errors (MLR) was used as estimator. The optimal full information maximum likelihood approach was used to estimate missing data (Collins et al., 2001). Each model fit to the data was estimated by examining multiple fit indices (Schermelleh-Engel et al., 2003), including the χ^2^ statistic, root mean square error of approximation (RMSEA), standardized root mean squared residual (SRMR) and the Bentler comparative fit index (CFI). The χ^2^ test was used to evaluate the appropriateness of the SEM model. The RMSEA measured the precision with which the covariances predicted by the model matched the actual covariances (approximate fit in the population). The RMSEA values ≤0.05 represented a good fit, values that were between 0.05 and 0.08 represented an adequate fit, values that were between 0.08 and 0.10 a mediocre fit, and values that were greater than 0.10 were inadmissible (Browne and Cudeck, 1993). The SRMR was the square root of averaged squared residuals (i.e., the differences between observed and predicted covariances). SRMR values <0.10 were acceptable. Nevertheless, a good fit was considered values that were less than 0.05 (Schermelleh-Engel et al., 2003). The CFI compared the covariance matrix that was predicted by the model with the observed covariance matrix and compared the null model with the observed covariance matrix. A CFI value greater than 0.97 indicates a good fit, whereas values greater than 0.95 represent an acceptable fit (Schermelleh-Engel et al., 2003). A SEM model was tested considering difficulties in ER and ability in WM latent variables as predictors. WM latent predictor has been tested on a previous study (Malagoli and Usai, 2018), whereas ER latent variables were based on an exploratory factor analysis (EFA) using principal axis factoring as the extraction method and varimax rotation of the factor structure. In the SEM model the YSR subscales were grouped into two latent variables representing internalizing and externalizing problems. As suggested by Achenbach and Rescorla (2001), the Anxious/Depressed, the Withdrawn/Depressed, and the Somatic Complaints subscales load on the internalizing factor, whereas the Rule-Breaking Behavior and the Aggressive Behavior subscales load on the externalizing factor. The scores of the remaining three subscales were entered in both the aforementioned latent factors.

## Results

The descriptive statistics are summarized in **Table 1**. Correlations among the measures are summarized in **Table 2**. All WM tasks show a pattern of significant correlations that remain when controlling for age. DERS subscales are significantly and moderately associated with each other, as are the YSR subscales. The pattern of associations between the different groups of measures is restricted to a few significant correlations. Considering the associations between the WM measures and the questionnaire subscales, the symmetry span task is negatively correlated with the DERS total score and with three DERS subscales: Lack of Emotional Awareness, Limited Access to ER Strategies, and Lack of Emotional Clarity. Moreover, the symmetry span task significantly correlates with the YSR - Aggressive Behavior subscale. The correlations between the symmetry span task and both the DERS - Lack of Emotional Awareness and the YSR – Aggressive Behavior subscales are not significant when controlling for age. Considering the pattern of associations between the questionnaires, the DERS – Difficulties Engaging in Goal-Directed Behavior is significantly associated with YSR – Somatic Complaints, and the DERS – Impulse Control Difficulties positively correlates with YSR – Social Problems, and these correlations remain significant when controlling for age.

**Table 1:** Descriptive statistics.

	Mean	SD	Skewness	SE	Kurtosis	SE
Symm_Span	17.44	7.947	0.328	0.169	-0.462	0.337
RSPAN	20.11	12.490	0.648	0.168	-0.090	0.335
Mr. Cucumber	6.46	1.159	-0.751	0.167	0.392	0.333
DERS_NonAcceptance	2.14	0.796	0.801	0.175	0.256	0.349
DERS_Goals	3.01	0.887	0.045	0.175	-0.513	0.349
DERS_Impulse	2.23	0.804	0.586	0.175	-0.377	0.349
DERS_Awareness	2.69	0.709	0.294	0.175	-0.419	0.349
DERS_Strategies	2.28	0.885	0.821	0.175	0.050	0.349
DERS_Clarity	2.35	0.786	0.743	0.175	-0.157	0.349
DERS_Total	87.43	20.305	0.400	0.175	-0.561	0.349
YSR_Anxious/Depressed	8.62	5.16	0.299	0.172	-0.598	0.342
YSR_Withdrawn/Depressed	4.75	3.19	0.581	0.172	-0.119	0.342
YSR_Somatic Complaints	4.96	3.42	0.503	0.172	-0.406	0.342
YSR_Social Problems	4.45	3.11	0.600	0.172	-0.426	0.342
YSR_Thought Problems	4.50	3.60	0.848	0.172	0.482	0.342
YSR_Attention Problems	6.58	3.33	0.116	0.172	-0.404	0.342
YSR_Rule Breaking	4.20	3.85	1.340	0.172	1.722	0.342
YSR_Aggressive_Behaviors	8.26	4.77	0.568	0.172	0.091	0.342

*Symm_Span = Symmetry complex span; RSPAN = Reading Span.*

**Table 2:** Zero order and partial correlation controlled for age (upper triangle).

	1	2	3	4	5	6	7	8	9	10	11	12	13	14	15	16	17	18
(1) Symm_Span	**-**	**0.246^∗∗^**	**0.196^∗∗^**	**-0.202^∗∗^**	-0.139	-0.136	-0.126	**-0.176^∗^**	**-0.212^∗∗^**	**-0.237^∗∗^**	0.055	0.050	0.115	0.069	0.086	0.053	0.114	0.144
(2) RSPAN	**0.250^∗∗^**	**-**	0.133	-0.038	-0.038	-0.112	-0.103	-0.013	-0.015	-0.073	-0.003	-0.050	0.098	-0.098	0.049	-0.023	0.059	0.100
(3) Mr. Cucumber	**0.208^∗∗^**	**0.139^∗^**	-	0.093	-0.059	-0.077	-0.019	-0.006	0.003	-0.015	-0.007	0.052	-0.056	0.032	0.049	0.026	-0.093	0.131
(4) DERS – Non-acceptance	**-0.198^∗∗^**	-0.037	0.095	**-**	**0.394^∗∗^**	**0.485^∗∗^**	0.045	**0.604^∗∗^**	**0.336^∗∗^**	**0.723^∗∗^**	0.097	0.094	0.120	0.046	0.007	0.042	0.046	0.020
(5) DERS - Goal	-0.139	-0.038	-0.059	**0.394^∗∗^**	-	**0.473^∗∗^**	0.052	**0.511^∗∗^**	**0.217^∗∗^**	**0.656^∗∗^**	0.041	0.050	**0.175^∗^**	0.115	0.046	0.145	0.061	0.057
(6) DERS – Impulse	-0.135	-0.112	-0.076	**0.485^∗∗^**	**0.473^∗∗^**	-	0.117	**0.565^∗∗^**	**0.342^∗∗^**	**0.744^∗∗^**	0.110	0.088	0.130	**0.159^∗^**	0.101	0.107	0.099	0.106
(7) DERS – Awareness	**-0.151^∗^**	-113	-0.049	0.037	0.051	0.113	-	**0.161^∗^**	**0.401^∗∗^**	**0.387^∗∗^**	0.014	0.049	-0.064	0.060	-0.041	-0.044	0.030	0.027
(8) DERS – Strategies	**-0.178^∗^**	-0.015	-0.009	**0.603^∗∗^**	**0.511^∗∗^**	**0.564^∗∗^**	**0.163^∗^**	-	**0.491^∗∗^**	**0.866^∗∗^**	0.060	0.035	0.086	0.037	-0.014	0.027	0.009	-0.041
(9) DERS – Clarity	**-0.227^∗∗^**	-0.024	-0.017	**0.327^∗∗^**	**0.214^∗∗^**	**0.338^∗∗^**	**0.423^∗∗^**	**0.489^∗∗^**	-	**0.654^∗∗^**	0.074	0.012	0.063	0.094	0.111	0.033	0.051	0.087
(10) DERS_Total	**-0.245^∗∗^**	-0.077	-0.026	**0.718^∗∗^**	**0.654^∗∗^**	**0.741^∗∗^**	**0.395^∗∗^**	**0.865^∗∗^**	**0.657^∗∗^**	-	0.096	0.078	0.127	0.117	0.044	0.074	0.067	0.050
(11) YSR-Anxious/Depressed	0.059	-0.001	-0.002	0.098	0.041	0.110	0.004	0.059	0.067	0.092	**-**	**0.680^∗∗^**	**0.575^∗∗^**	**0.692^∗∗^**	**0.526^∗∗^**	**0.485^∗∗^**	**0.243^∗∗^**	**0.435^∗∗^**
(12) YSR – Withdrawn/Depressed	0.064	-0.043	0.067	0.097	0.049	0.087	0.016	0.031	-0.009	0.066	**0.679^∗∗^**	-	**0.454^∗∗^**	**0.647^∗∗^**	**0.380^∗∗^**	**0.354^∗∗^**	**0.323^∗∗^**	**0.324^∗∗^**
(13) YSR - Somatic Complaints	0.122	0.102	-0.046	0.121	**0.175^∗^**	0.130	-0.079	0.084	0.051	0.120	**0.576^∗∗^**	**0.458^∗∗^**	-	**0.453^∗∗^**	**0.476^∗∗^**	**0.413^∗∗^**	**0.373^∗∗^**	**0.331^∗∗^**
(14) YSR – Social Problems	0.071	-0.096	0.035	0.047	0.115	**0.159^∗^**	0.053	0.036	0.089	0.114	**0.692^∗∗^**	**0.644^∗∗^**	**0.454^∗∗^**	-	**0.479^∗∗^**	**0.505^∗∗^**	**0.374^∗∗^**	**0.561^∗∗^**
(15) YSR – Thought Problems	0.091	0.052	0.055	0.009	0.046	0.100	-0.052	-0.016	0.102	0.039	**0.527^∗∗^**	**0.383^∗∗^**	**0.478^∗∗^**	**0.480^∗∗^**	-	**0.493^∗∗^**	**0.424^∗∗^**	**0.537^∗∗^**
(16) YSR – Attention Problems	0.062	-0.019	0.036	0.044	0.144	0.106	-0.063	0.025	0.020	0.067	**0.487^∗∗^**	**0.360^∗∗^**	**0.416^∗∗^**	**0.505^∗∗^**	**0.494^∗∗^**	-	**0.470^∗∗^**	**0.510^∗∗^**
(17) YSR – Rule-Breaking Behavior	0.128	0.066	-0.075	0.049	0.060	0.098	-0.003	0.006	0.029	0.055	**0.246^∗∗^**	**0.334^∗∗^**	**0.378^∗∗^**	**0.374^∗∗^**	**0.427^∗∗^**	**0.475^∗∗^**	-	**0.522^∗∗^**
(18) YSR - Aggressive Behavior	**0.153^∗^**	0.104	0.141	0.022	0.056	0.106	0.004	-0.044	0.071	0.042	**0.436^∗∗^**	**0.331^∗∗^**	**0.335^∗∗^**	**0.560^∗∗^**	**0.538^∗∗^**	**0.514^∗∗^**	**0.527^∗∗^**	-
Age in Months	0.119	0.055	0.127	0.028	-0.003	0.000	**0.245^∗∗^**	-0.028	**-0.161^∗^**	-0.086	0.037	0.126	0.069	0.023	0.049	0.081	0.129	0.090
Gender	0.113	0.011	-0.065	0.121	0.039	0.134	0.067	**0.173^∗^**	**0.377^∗∗^**	**0.216^∗∗^**	0.125	0.050	**0.190^∗∗^**	0.077	-0.018	-0.068	-0.097	-0.031

*^∗^Correlation is significant at 0.05 (2-tails). ^∗∗^Correlation is significant at 0.01 (2-tails). Symm_Span = Symmetry complex span; RSPAN = Reading Span.*

The EFA extracted two factors that account for 51% of the total variance in the DERS. The DERS subscales load mainly on factor 1 (35% of the total variance): Non-acceptance (factor loading = 0.686), Goals (factor loading = 0.620), Impulse (factor loading = 0.686), and Strategies (factor loading = 0.801). Awareness and Clarity subscales load on factor 2 (16%; factor loadings 0.554 and 0.755, respectively). The two factors are labeled difficulties in emotion response (EM_R) and difficulties in emotion knowledge (EM_K), respectively. Indeed, factor 1 (EM_R) consisted of subscales measuring the difficulties in managing a response to an emotional elicitation. Factor 2 (EM_K) included two subscales measuring the difficulties in understanding an emotional state.

**FIGURE 1:**
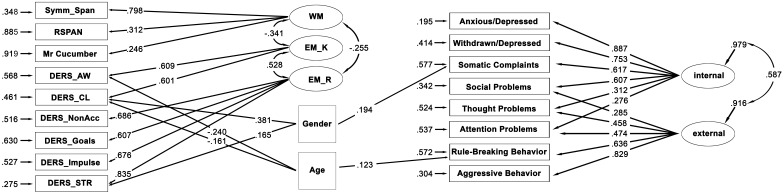
The SEM model representing the significant relationships between WM, EM_K and EM_R latent factors with behavioral outcomes. The ellipses represent the latent variables, and the rectangles represent the individual tasks or questionnaires scales (manifest variables). The curved double-headed arrows represent correlations among the latent variables. The straight, single-headed arrows refer to significant associations. The standardized factor loadings are the numbers next to the straight, single-headed arrows. The error terms are shown near the observed variables at the end of the smaller, single-headed arrows. EM_K = Emotion Regulation Knowledge; EM_R = Emotion Regulation Response; WM = Working Memory; Symm_Span = Symmetry complex span; RSPAN = Reading Span; Internal = Internalizing behaviors; External = Externalizing behaviors.

Considering the SEM analysis, a full factorial model controlled for age and gender, with three latent variables as predictors and two latent factors as outcomes representing internalizing and externalizing problems, is tested. The error-term squares were considered to be estimates of the unexplained variance for each measure. The fit indices were good or acceptable, excepting for the chi square, but this statistic is very sensitive to sample sizes, and in case of large sample size (greater than 200), authors suggest relying upon other indices (Schermelleh-Engel et al., 2003): χ^2^ = 165.447, *df* = 107, *p* = 0.000, RMSEA = 0.049 [90% CI = 0.034–0.063], SRMR = 0.053, and CFI = 0.947. The model complete with the standardized solution is illustrated in **Figure 1** and the parameters are shown in **Table 3**. Two out six DERS subscales, i.e., Awareness and Clarity, load on a latent variable named difficulties in ER knowledge (EM_K). The other four DERS subscales (Non-acceptance, Goals, Impulse, and Strategies) load on a latent variable named difficulties in ER response (EM_R). The three WM measures load on the latent variable WM. The factor loadings for these latent variables are all significant (*t*-values >2, **Table 3**). The three latent predictors correlate with each other. The eight YSR measures load on the two Internalizing and Externalizing latent factors (*t*-values >2, **Table 3**). The EM_R, the EM_K, and the WM latent variables are considered as predictors of the two YSR latent factors, but none of these contribute significantly. The model shows that difficulties in ER are significantly associated with WM. The factor loading is negative, indicating that high levels of ER difficulties on knowledge and response are associated with low WM ability.

**Table 3:** Factor model parameters.

		Estimate	S.E.	Est./S.E.	*P*-value
WM BY	Symm_Span	0.798	0.218	3.653	0.000
	RSPAN	0.312	0.131	2.375	0.018
	Mr. Cucumber	0.246	0.083	2.967	0.003
EM_R BY	DERS - Non-acceptance	0.686	0.053	12.905	0.000
	DERS - Goal	0.607	0.056	10.842	0.000
	DERS - Impulse	0.676	0.055	12.301	0.000
	DERS - Strategies	0.835	0.052	16.171	0.000
EM_K BY	DERS - Awareness	0.609	0.043	14.089	0.000
	DERS - Clarity	0.601	0.047	12.739	0.000
WM WITH	EM_R	-0.255	0.095	-2.690	0.007
WM WITH	EM_K	-0.341	0.131	-2.596	0.009
EM_K WITH	EM_R	0.528	0.095	5.538	0.000
Internalizing by	YSR - Anxious/Depressed	0.887	0.028	31.136	0.000
	YSR - Withdrawn/Depressed	0.753	0.036	20.761	0.000
	YSR - Somatic Complains	0.617	0.048	12.979	0.000
	YSR - Social Problems	0.607	0.070	8.632	0.000
	YSR - Thought Problems	0.312	0.087	3.576	0.000
	YSR - Attention Problems	0.276	0.090	3.073	0.002
Externalizing by	YSR - Rule-Breaking	0.636	0.054	11.830	0.000
	YSR - Aggressive Behaviors	0.829	0.056	14.771	0.000
	YSR - Social Problems	0.285	0.081	3.535	0.000
	YSR - Thought Problems	0.458	0.094	4.897	0.000
	YSR - Attention Problems	0.474	0.099	4.797	0.000
Internalizing ON	WM	0.105	0.098	1.065	0.287
	EM_R	0.136	0.111	1.221	0.222
	EM_K	-0.022	0.138	-0.162	0.871
Externalizing ON	WM	0.276	0.151	1.830	0.067
	EM_R	0.015	0.136	0.111	0.911
	EM_K	0.217	0.160	1.349	0.177
Symm_Span ON	Age	0.123	0.072	1.717	0.086
	Gender	0.029	0.069	0.415	0.678
RSPAN ON	Age	0.068	0.072	0.953	0.340
	Gender	0.120	0.064	1.885	0.059
Mr. Cucumber ON	Age	0.130	0.071	1.822	0.068
	Gender	-0.058	0.064	-0.905	0.365
DERS – Non-acceptance ON	Age	0.028	0.071	0.385	0.700
	Gender	0.118	0.069	1.708	0.088
DERS – Goal ON	Age	-0.008	0.084	-0.090	0.928
	Gender	0.034	0.071	0.485	0.628
DERS – Impulse ON	Age	0.000	0.075	0.001	0.999
	Gender	0.129	0.070	1.834	0.067
DERS – Awareness ON	Age	-0.240	0.066	-3.614	0.000
	Gender	0.047	0.062	0.753	0.451
DERS – Strategies ON	Age	-0.028	0.070	-0.398	0.691
	Gender	0.165	0.071	2.340	0.019
DERS – Clarity ON	Age	-0.161	0.066	-2.447	0.014
	Gender	0.381	0.052	7.269	0.000
YSR – Anxious/Depressed ON	Age	0.044	0.073	0.601	0.548
	Gender	0.128	0.070	1.830	0.067
YSR – Withdrawn/Depressed ON	Age	0.129	0.075	1.717	0.086
	Gender	0.058	0.068	0.844	0.399
YSR – Somatic Complains ON	Age	0.079	0.065	1.204	0.228
	Gender	0.194	0.064	3.047	0.002
YSR – Social Problems ON	Age	0.028	0.069	0.401	0.689
	Gender	0.083	0.070	1.182	0.237
YSR – Thought Problems ON	Age	0.048	0.068	0.701	0.483
	Gender	-0.010	0.070	-0.139	0.890
YSR – Attention Problems ON	Age	0.077	0.068	1.136	0.256
	Gender	-0.058	0.070	-0.828	0.408
YSR – Rule-Breaking ON	Age	0.123	0.057	2.169	0.030
	Gender	-0.084	0.072	-1.179	0.238
YSR – Aggressive Behaviors ON	Age	0.087	0.065	1.344	0.179
	Gender	-0.018	0.072	-0.249	0.804

*Uppercase words in the first column refer to the M-Plus sintax: BY relates latent factors with their observed variables; ON means that a variable is predicted from one or more others latent or observed variables; WITH indicates a correlation between variables; Symm_Span = Symmetry complex span; RSPAN = Reading Span.*

Age and gender influenced significantly a few measures. Higher Awareness and Clarity difficulties were shown by the youngest individuals (and vice-versa), that also tend to report less problems on the Rule-Breaking Behavior YSR subscale. As regard to gender influences, females reported more difficulties on Clarity and Strategies DERS subscales and more Somatic Complaints in the YSR self-report.

## Discussion

This study investigates the relationships between difficulties in ER, WM and self-reported aspects of behavioral outcomes in typically developing adolescents and young adults. In particular, it considers the cognitive features of ER and WM, and this study examines if these components of self-regulation can be associated with non-adaptive outcomes in a typically developed population.

### Analysis of Correlations: Pattern in WM Tasks, DERS and YSR

A preliminary Pearson correlation analysis indicated that all WM tasks were correlated among themselves, as were DERS and YRS sub-scales. Pearson correlations also showed a negative correlation between the symmetry span task, the DERS total score and three DERS subscales: Lack of Emotional Awareness, Limited Access to ER Strategies and Lack of Emotional Clarity. These relationships among visual WM and specific difficulties in awareness, strategies and clarity, are particularly interesting considering the power that emotion has both to fix information (Jones et al., 1987; Dietrich et al., 2001; Schmidt and Williams, 2001) or to interfere with the recall of it (Dolcos and McCarthy, 2006; Dolcos et al., 2008; Anticevic et al., 2010; Chuah et al., 2010; Denkova et al., 2010). The symmetry span task significantly correlates also with the YSR – Aggressive Behavior subscale, but this association, as well the association with the DERS – Lack of Emotional Awareness subscale, was no longer significant when controlling for age.

Considering now the pattern of association between the DERS scale and the YSR self-report, the DERS – Difficulties Engaging in Goal-Directed Behavior is significantly associated with YSR – Somatic Complaints, and the DERS – Impulse Control Difficulties positively correlates with YSR – Social Problems, and these correlations remain significant when controlling for age. These associations seem to show an actual connection, specifically between more visual aspects of WM and specific difficulties in ER and behavioral outcomes, that mostly maintain their significance even controlling for age, as if this pattern may be somehow typical of the age range. Given these relationships, the SEM analysis enabled us to investigate this existing relationship better considering latent variables and possibly different outcomes.

### Association Between WM and ER Components

The SEM analysis considers three latent variables representing difficulties in ER knowledge (EM_K) and in ER response (EM_R) perceived by the participants loaded by the DERS scales and a unitary latent dimension for WM abilities. This dual organization for ER variables is coherent with the process model of ER abilities illustrated by Gross and Thompson (2007): emotion-generative process vs. cognitive reappraisal, which in our model would be reflected by EM_R factor and EM_K factor respectively. The unitary WM model as well has already been documented by existing literature (e.g., Huizinga et al., 2006; McAuley and White, 2011) and this specific one has been tested in a previous study meant to investigate the organization of WM and inhibition during adolescence (Malagoli and Usai, 2018).

Age and gender show limited influence and in accordance with the literature. With age increasing, the perceived difficulties in the understanding of mental states related to an emotion decrease (Zimmermann and Iwanski, 2014). Females more than males, in addition to reporting difficulties in clearly perceiving emotions, tend to report more problems in ER strategies (Weinberg and Klonsky, 2009). Moreover, females report more frequent somatic problems (Rescorla et al., 2007). After controlling for the aforementioned age and gender influences, the model shows that WM is negatively associated with both dimensions tapping ER difficulties.

This negative relationship may be interpreted on one hand as the awareness that emotional difficulties interfere with WM abilities while, on the other hand, as better WM abilities may reduce the impact of ER difficulties. This result is coherent with the literature on adults and with theories and neuro-studies on adolescents that show an association between emotional components, such as difficulties in managing emotions, and more cognitive abilities such as WM, as they could actually interfere or modulate each other (Dahl, 2004; Dolcos and McCarthy, 2006; Dolcos et al., 2008; Anticevic et al., 2010; Chuah et al., 2010; Denkova et al., 2010; Bridgett et al., 2013; Hendricks and Buchanan, 2016). Bridgett et al. (2013), in particular, investigating the effects of EF, and particularly complex features of WM (updating and monitoring) toward more specific aspects of self-regulation, such as effortful control, show how all these aspects may be differentiated versus integrated in explaining the role of self-regulatory systems in ER.

The literature documents a slower development of subcortical versus dorsal brain regions, and often this slower development has been considered one possible explanation for risk-taking and difficulties in managing more emotional situations, as this difference in time could interfere with cognitive processing of information (Giedd et al., 1996, 1999; Sowell et al., 1999; Dahl, 2004; Gogtay et al., 2004; Hooper et al., 2004; Barnea-Goraly et al., 2005). An interesting perspective that could explain this condition is given by Lewis and Todd (2007). In fact, in the authors’ theorization, they reflect on how it can be highly difficult and tricky to speak separately about cognitive regulation and emotional regulation. On one side, it is clear that certain forms of regulation are carried out by executive processes, top-down and more subject to voluntary control, while others seem to click in a more “automatic” way and rely on more primitive processes. The key point is that these processes are in constant interaction, and this interaction increases activity that can be both cognitive and emotional. Using the concept of neuroaxis, Lewis and Todd (2007) analyzed the brain activity in a bidirectional way. They imagined these processes as the extremes of a vertical continuum that can be “walked through” both from top-down to bottom-up and vice-versa. In this sense, the system that may have a larger impact on decision-making processes may change according to the situation, and in particular emotional situations, and behavior may be driven primarily by processes that are more primitive and less influenced by the environment, similar to control processes.

### Behavioral Outcomes, ER and WM: Which Relationship?

Internalizing/externalizing factors of YSR appear to be separate yet related coherently with the latent organization suggested by Achenbach and Rescorla (2001), although no significant and direct relation with ER and WM factor appeared. Our results, in fact, show how difficulties in emotion regulation are associated with WM efficiency, with WM that appear to be affected by more complex aspects of regulation. This specific result is particularly interesting, especially considering the age range investigated and the potential in terms of adding knowledge about the connection between two dimensions often treated as separate such as emotion regulation and WM, in a time of enhancement of the emotional arousal and growth of cognitive skills. On the other hand the absence of significant direct relation between WM and YSR may represent a less consistent result with respect to the documented relation between WM and the two ER factors. One possible explanation calls into account the problematic use of the YSR scale with the typically developing population. In fact, the presence of much-polarized items may not reflect a typical expression of internalizing or externalizing tendencies, thus the YSR scale may be more useful to discriminate extreme behaviors than to capture individual differences within the continuum of the norm.

Also, the fact that our sample is not a clinical one may have a role in not finding a strong association between these components. While presenting results, most participants indicated that they actually could recognize themselves in Dahl’s paradox, meaning they sometimes could not help their feelings and emotions that they felt “all over the place.” Experiencing emotions and actually mentalising about them may be two very different issues, especially during adolescence (Dahl, 2004), and the emotional impact of the event may highlight these episodes in their minds so they report them due to their vivid memory of it. With regard to the organization of ER, behavioral outcomes and WM, there is a general lack of studies that directly document similar results in terms of WM efficiency and its relation with specific self-reported aspects of regulation, both behavioral and emotional.

Another possible explanation with reference to this specific result, may be found in the hypothesis formulated by Romer et al. (2009), who found non-direct relation between cognitive features and some more complex outcomes related also to more emotional issues may exist In fact, according to this hypothesis, in regression analysis impulsivity seems to be related to externalizing behavior whereas WM shows only indirect association with externalizing behavior mediated by impulsivity and more complex aspects of human behavior such as sensation seeking. We are not able to demonstrate that in our sample as we did not consider sensation seeking for our study although this interpretation would make sense as WM and Inhibition work together in managing complex everyday situations (Diamond, 2013) and would be an interesting perspective to be considered for future research. In conclusion, the results of the present study confirm the importance of ER difficulties in WM and in being a possible explanation for individual differences. In turn, WM is important in managing the impact of emotional elicitations but also in enhancing the awareness of difficulties, in reporting more aggressive behaviors. From this perspective, the awareness from having strong WM may be used as a tool for intervention in the TD population, who is in a delicate time of growth (Crone, 2009; Dahl, 2004), to be able to help them to cope with these statistically normal difficulties and prevent future discomfort or disease.

### Limitations to the Present Study

Several limitations of this study warrant mentioning. We used self-reported questionnaires basically developed as screening instruments that investigate social aspects of regulation indirectly, such as tendency to “lose control” or “getting into a fight,” which are specific and social aspects at one extreme, while such phenomena as “going blank” and not being able to complete a task even when the rules to perform it have already been learned but emotional arousal or anxiety prevent the individual from accessing that knowledge, were not investigated. Additionally, a debriefing asking in order to better understand the three-point responses to the YSR would have been useful. Despite these limitations, the study showed some strengths, taking into account a developmental period that has been less investigated, and comparing it with adulthood and childhood using a larger and more uniform sample, in terms of age range, than those that have been employed in previous studies (e.g., Romer et al., 2009, 2011). It also offers an investigation of less explored aspects of the existing relationship between WM, ER and behavioral outcomes.

## Conclusion

In general, this study showed a significant relationship between self-reported difficulties in ER and WM, adding knowledge regarding how behavioral and emotional self-reported outcomes may relate to these processes.

## Ethics Statement

This study was carried out in accordance with the recommendations of the Ethical Code of Italian Psychology Order and of the Ethical guidelines of the Italian Association of Psychology with written informed consent from all subjects. All parents of the subjects gave written informed consent in accordance with the Declaration of Helsinki. At the time we collected the data no ethical committee was yet present to which we could refer to.

## Author Contributions

CM and MCU revised the literature and conceived and designed the study. CM collected the data. MCU run the analysis. CM wrote the first draft of the manuscript, that was revised also by MCU. All authors read and approved the final manuscript.

## Conflict of Interest Statement

The authors declare that the research was conducted in the absence of any commercial or financial relationships that could be construed as a potential conflict of interest.
